# Imaging tumour cell heterogeneity following cell transplantation into optically clear immune-deficient zebrafish

**DOI:** 10.1038/ncomms10358

**Published:** 2016-01-21

**Authors:** Qin Tang, John C. Moore, Myron S. Ignatius, Inês M. Tenente, Madeline N. Hayes, Elaine G. Garcia, Nora Torres Yordán, Caitlin Bourque, Shuning He, Jessica S. Blackburn, A. Thomas Look, Yariv Houvras, David M. Langenau

**Affiliations:** 1 Molecular Pathology, Cancer Center, and Regenerative Medicine, Massachusetts General Hospital, Boston, Massachusetts 02129, USA; 2 Harvard Stem Cell Institute, Harvard University, Cambridge, Massachusetts 02139, USA; 3 Abel Salazar Biomedical Sciences Institute, University of Porto, Porto 4099-003, Portugal; 4 Department of Surgery, Weill Cornell Medical College, New York, New York 10065, USA; 5 Department of Medicine, Weill Cornell Medical College, New York, New York 10065, USA; 6 Pediatric Oncology, Dana-Farber Cancer Institute, Boston, Massachusetts 02115, USA; 7 Department of Molecular and Cellular Biochemistry, University of Kentucky, Lexington, Kentucky 40506, USA

## Abstract

Cancers contain a wide diversity of cell types that are defined by differentiation states, genetic mutations and altered epigenetic programmes that impart functional diversity to individual cells. Elevated tumour cell heterogeneity is linked with progression, therapy resistance and relapse. Yet, imaging of tumour cell heterogeneity and the hallmarks of cancer has been a technical and biological challenge. Here we develop optically clear immune-compromised *rag2*^*E450fs*^ *(casper)* zebrafish for optimized cell transplantation and direct visualization of fluorescently labelled cancer cells at single-cell resolution. Tumour engraftment permits dynamic imaging of neovascularization, niche partitioning of tumour-propagating cells in embryonal rhabdomyosarcoma, emergence of clonal dominance in T-cell acute lymphoblastic leukaemia and tumour evolution resulting in elevated growth and metastasis in *BRAF*^*V600E*^-driven melanoma. Cell transplantation approaches using optically clear immune-compromised zebrafish provide unique opportunities to uncover biology underlying cancer and to dynamically visualize cancer processes at single-cell resolution *in vivo.*

## Abstract (web summary)

Direct visualisation of heterogeneous cell populations in live animals has been challenging. Here, the authors optimize cell transplantation into optically clear immune-deficient zebrafish, and use intravital imaging to track and to assess functional diversity of individual cancer cells *in vivo*.

## Graphical abstract

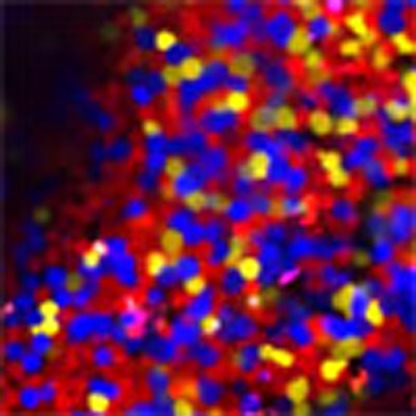

The conversion of phenotypically normal cells into malignant cells is often associated with acquired ‘hallmarks of cancer' including elevated growth potential, suppression of cell death pathways, development of new vascular networks to feed the growing tumour and acquired cell motility that lead to invasion and metastasis^1^,^2^. Despite our increased understanding that cancer is driven by molecular changes that convert normal cells into malignant cells, it has become well recognized that not all cancer cells are created equal. For example, the process of clonal evolution that is responsible for initiating cancer is also a critical driver of intra-tumoral heterogeneity that is constantly arising throughout the lifespan of a cancer cell^3^. This heterogeneity provides a rich diversity of cell types and mutations on which natural selection can act, ultimately leading to a subset of cancers that elevate metastatic potential and therapy resistance^4^,^5^,^6^. Despite our new found ability to genetically map individual mutations that are acquired during tumour cell evolution and progression^7^,^8^,^9^, it is been difficult to directly visualize how these mutations affect tumour growth in live animals. Access to optically clear animal models would permit the dynamic visualization of the cancer hallmarks and provide unprecedented access to dissect the molecular underpinnings of cancer progression at single-cell resolution.

Over the past two decades, the field of cancer research has been empowered by intra-vital imaging (IVM) in mouse models^10^,^11^. Refined imaging tools, including confocal, multi-photon and light sheet microscopy, have now been applied to a wide range of cancers to facilitate the discovery of underlying mechanisms that drive cancer growth *in vivo*. For example, Kedrin *et al.* have utilized photoactivatable fluorescent proteins and cell lineage tracing to visualize the tumour cell niche in mammary carcinoma. These studies uncovered that cells adjacent to the vasculature drive invasion and metastasis^12^. Using similar approaches, Calabrese *et al.* discovered that Nestin+ brain tumour stem cells reside in a perivascular niche^13^. Metastasis has also been observed using IVM. For example, a single extravasated C26 colorectal cancer cell has been shown to proliferate and to produce highly mobile pre-metastatic lesions in the liver^14^. Sophisticated fluorescent labelling techniques, including cell lineage tracing using Confetti and brainbow constructs^15^,^16^, have now been successfully integrated with high-resolution microscopy to visually dissect intra-tumoral heterogeneity. For example, Zomer *et al.* utilized the Confetti strategy to label individual mammary tumour cells, and performed proof-of-concept experiments to show that tumour stem cells can become alternatively active or quiescent during tumour development^17^. Despite these successes, challenges presented by the opacity of furred rodents, and the requirement for invasive surgical implantation of imaging windows have limited the application of IVM. Furthermore, requirements of imaging through pre-defined windows often prohibit simultaneous observation of tumour cells from the primary and metastatic sites within the same animal.

Zebrafish have been developed as a robust model of human cancer and have now been widely used for visualizing cancer processes in live animals. For example, our group has used fluorescent transgenic approaches to label embryonal rhabdomyosarcoma (ERMS) cells based on differentiation status. Using these approaches, we have been able to dynamically visualize tumour cell heterogeneity *in vivo*, identifying the existence of a molecularly defined tumour-propagating cell (TPC) that expresses myf5-GFP and other differentiated cell types that express myogenin and drive invasion^18^. Others have utilized cell transplantation into irradiated, optically clear *casper* strain adult zebrafish to visualize melanoma invasion^19^, and conversion of T-lymphoblastic lymphoma into leukaemia^20^. Importantly, these initial successes utilized cell transplantation into either syngeneic strains of zebrafish or irradiated recipient animals that only transiently dampen immune responses. Using genome-editing approaches^21^, our group has recently developed homozygous *rag2*^*E450fs*^ zebrafish. These fish are viable as adults, have deficiencies in T and B cells, and enable robust engraftment of fluorescently labelled zebrafish tumour cells from a wide range of cancers and strains of zebrafish^22^. Despite the utility of the *rag2*^*E450fs*^ model for cell engraftment studies, the *rag2*^*E450fs*^ mutation was created on the pigmented AB-strain and thus it has been difficult to image tumour cells at single-cell resolution in engrafted animals.

Here we create transparent *casper* strain, *rag2*^*E450fs*^ mutant zebrafish and utilize these animals for transplantation studies to image heterogeneity and various ‘hallmarks of cancer'. For example, confocal imaging permits the dynamic visualization of TPCs in ERMS and the emergence of clonal dominance in T-cell acute lymphoblastic leukaemia (T-ALL). Serial imaging studies also detail the evolution of metastasis in a subset of *BRAF*^*V600E*^-driven melanomas and facilitate the direct visualization of micro-metastatic disease. Our work provides a universal transplantation model for imaging cancer cell processes, opening new avenues for visualizing the functional consequences of cancer cell heterogeneity and clonal evolution at single-cell resolution in the zebrafish.

## Results

### Engraftment of allogeneic tumours

**Figure 1:**
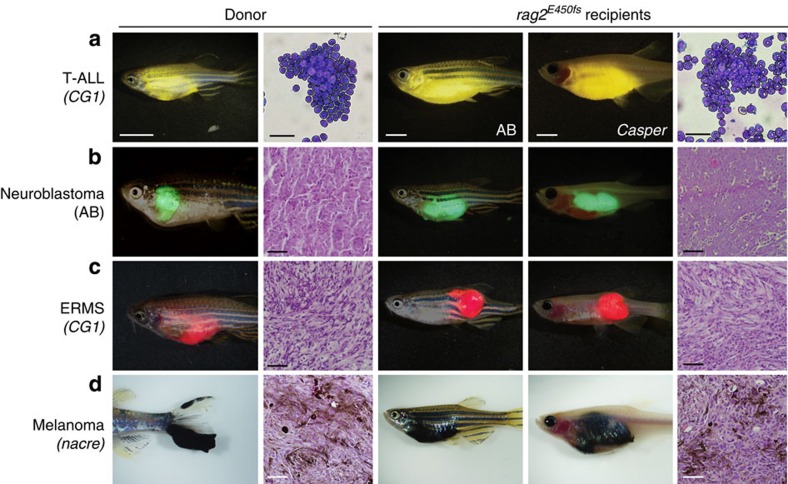
Zebrafish cancers engraft into homozygous *rag2*^*E450fs*^ *(casper)* animals. Donor animals shown in the left two panels while transplant recipients are to the right. (**a**) ZsYellow-labelled *Myc*-driven T-ALL from the syngeneic *CG1* background, (**b**) EGFP-labelled neuroblastoma from AB background, (**c**) mCherry-labelled *kRAS*^*G12D*^*-*driven ERMS from *CG1* background, and (**d**) *BRAF*^*V600E*^-induced melanoma arising in *tp53*^*−/−*^*nacre* background. Tumour cells were transplanted intra-peritoneally (**a**,**b**,**d**) or intra-muscularly (**c**) into both *rag2*^*E450fs*^ (AB) and *rag2*^*E450fs*^ *(casper)-*recipient fish. Merged brightfield and fluorescent images are shown at 30 d.p.t. Cytospins of leukaemia cells are shown in **a**, whereas haematoxylin and eosin (H&E)-stained sections are shown in **b**–**d**. Scale bars equal 5 mm in whole animals images, 20 μm for cytospins shown in **a**, and 50 μm for histology sections shown in **b**–**d**.

We have previously reported the production of lymphocyte-deficient *rag2*^*E450fs*^ mutant zebrafish that engraft a wide variety of normal and malignant zebrafish cells^22^ (ZFIN allele *rag2*^*fb101*^). However, these initial studies utilized pigmented, AB-strain zebrafish, making it difficult to directly visualize tumour cells at high resolution *in vivo*. To facilitate imaging of cancer in live zebrafish, the *rag2*^*E450fs*^ mutation was bred into the *casper* background—a transparent zebrafish that lacks melanocytes and iridophores^19^. As expected, *rag2*^*E450fs*^ (*casper*) zebrafish efficiently engrafted fluorescently labelled T-ALL^23^, neuroblastoma^24^, ERMS^18^,^25^ and melanoma^26^,^27^ (Fig. 1, Supplementary Fig. 1 and Supplementary Table 1). Tumours derived from *CG1*, AB and *nacre* strain zebrafish engrafted efficiently into *rag2*^*E450fs*^ *(casper)* zebrafish and did not require matching at the major histocompatibility complex or pre-conditioning with γ-irradiation. Importantly, engrafted tumours exhibited similar histology as donor tumours (Fig. 1). As has been reported previously for AB-strain wild-type transplant recipients^22^, tumours failed to engraft into unconditioned *casper-*strain animals that have an intact rag2 locus (Supplementary Table 1 and Supplementary Fig. 1).

### Visualizing the dynamics of tumour neovascularization

**Figure 2:**
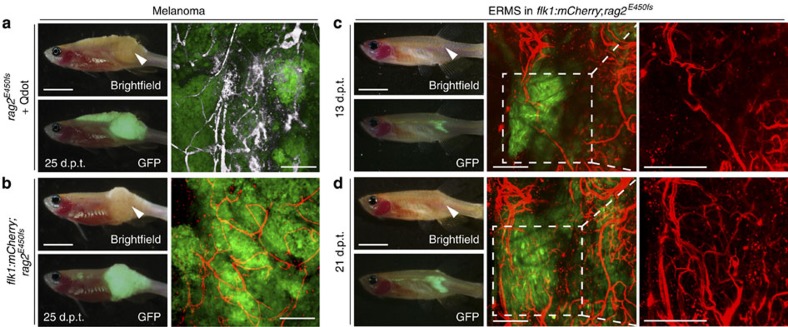
Imaging neovascularization in *rag2*^*E450fs*^ *(casper)* zebrafish engrafted with fluorescently labelled melanoma and ERMS. (**a**) GFP-labelled, amelanotic melanoma implanted into *rag2*^*E450fs*^ *(casper)* fish (*n*=8 animals) and imaged following intravascular injection of crimson quantum dots (Qtracker 655). Whole animal images to the left and confocal images to the right ( × 100 magnification, 100–200 μm z-stack). Quantum dot fluorescence has been pseudo-coloured white. (**b**) GFP-labelled, amelanotic melanoma implanted into *flk1:mCherry; rag2*^*E450fs*^ *(casper)* transgenic zebrafish (*n*=4 animals). (**c**–**d**) GFP-labelled ERMS engrafted into *flk1:mCherry; rag2*^*E450fs*^ *(casper)* transgenic zebrafish (*n*=5 animals) and serially imaged over time (**c**, 13 d.p.t. and **d**, 21 d.p.t.). White arrowheads denote the site of intra-muscular injection of tumour cells. Scale bars equal 5 mm in whole animal images and 200 μm in confocal images.

Neovascularization is an important hallmark of cancer and has been imaged in a variety of cancers^18^,^28^,^29^. To assess the utility of adult *rag2*^*E450fs*^ *(casper)* fish for imaging neovascularization in the transplantation setting, green fluorescent protein (GFP)-labelled *BRAF*^*V600E*^*, tp53*^*−/−*^ amelanotic melanoma^27^ cells were implanted into the dorsal musculature of 3-month-old *rag2*^*E450fs*^ *(casper)* fish (5 × 10^5^ cells in 2 μl per animal). Because engraftment was initially limited to the dorsal musculature, melanomas developed adjacent to the skin epidermis. Neovascularization could be directly visualized by confocal microscopy following injection of crimson quantum dots^30^ into the blood stream at 25 days post transplantation (d.p.t.; Fig. 2a, *n*=8 animals). Crimson quantum dots were chosen because they excite in far-red wavelengths and can be easily differentiated from other fluorescent proteins used to label tumour cells in this study, including AmCyan, GFP, ZsYellow, DsRED and mCherry. To further refine imaging of neovascularization within solid tumours, we next created *flk1:mCherry*; *rag2*^*E450fs*^ *(casper)* fish with fluorescently labelled vasculature. Animals were injected intra-muscularly with GFP-labelled melanoma (5 × 10^5^ cells in 2 μl per animal). Following successful engraftment at the site of injection, vessels were readily visualized by confocal microscopy ( × 100 magnification, 105.71 μm=10 z-stacks, *n*=4 animals, Fig. 2b). Finally, we have been able to visualize how neovascularization changes over time within the same animal, which results in the creation of dense vascular networks associated with ERMS growth ( × 100 magnification, 223.64 μm=10 z-stacks, Fig. 2c,d). Together, these experiments demonstrate the ease and utility of imaging neovascularization in solid tumours using the *rag2*^*E450fs*^ *(casper*) fish.

### Imaging ERMS heterogeneity at single-cell resolution

**Figure 3:**
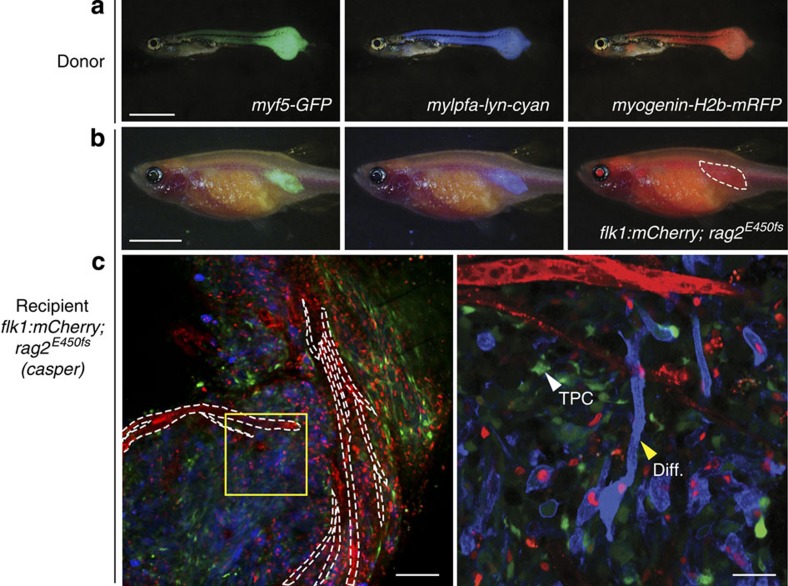
Resolving tumour cell heterogeneity in ERMS at single-cell resolution following engraftment into *flk1:mCherry; rag2*^*E450fs*^ *(casper)* zebrafish. (**a**) Epi-fluorescent images of primary ERMS in a 32-day-old *myf5:GFP; myogenin-H2b:mRFP; mylpfa:lyn-cyan* triple transgenic zebrafish. (**b**) Epi-fluorescent images of *flk1:mCherry; rag2*^*E450fs*^ *(casper)-*recipient fish engrafted intra-muscularly with fluorescently labelled ERMS at 28 d.p.t. (*n*=4 animals). (**c**) Confocal image with mCherry-labelled vasculature outlined by white dashed lines (left, × 100 magnification). Higher magnification of boxed region (right, × 400 magnification). Myosin-expressing, differentiated cells (Diff.) and less frequent *myf5-GFP+* tumour-propagating cell (TPC) denoted by arrowheads. Scale bar equals 2 mm in **a**, 5 mm in **b**, 100 μm (**c**, left panel) and 25 μm (**c**, right panel).

We next wanted to dynamically visualize tumour cell heterogeneity at single-cell resolution using a transgenic model of *kRAS*^*G12D*^-driven ERMS^25^. We have previously created primary ERMS in triple transgenic *myf5:GFP; myogenin-H2b:mRFP; mylpfa:lyn-cyan* zebrafish, which enables the labelling of tumour cells based on differentiation status^18^. To achieve direct imaging of heterogeneous tumour cell populations in adult zebrafish, we engrafted these fluorescent transgenic ERMS by intra-muscular injection into 3-month-old adult *flk1:mCherry*; *rag2*^*E450fs*^ *(casper)* strain zebrafish (4 × 10^5^ cells in 2 μl per animal, Fig. 3a,b). Confocal microscopic imaging revealed that GFP-labelled TPCs and AmCyan-labelled differentiated cells were largely confined to distinct regions of the tumour ( × 100 magnification, Fig. 3c, left panel), consistent with previous reports^18^. Remarkably, the vasculature transects areas of regional tumour cell heterogeneity. We have also noted that in this particular case, juxtaposition of specific ERMS cell subtypes near vascular beds was not observed. Higher magnification imaging of areas enriched with differentiated ERMS cells revealed the presence of *myf5-GFP+* TPCs, albeit at reduced numbers when compared with other regions of the tumour ( × 400 magnification, Fig. 3c, right panel). Our data suggest that ERMS cells largely reside in regionally defined niches based on differentiation status and are not anatomically confined by proximity to vessels. The combination of fluorescent transgenic labelling of tumour cell subpopulations and subsequent cell transplantation will be important for defining how niche topology is established and ultimately influences continued tumour growth in ERMS and a wide range of cancers.

### Detailing emergence of clonal dominance in T-ALL

We next wanted to use our model to dynamically visualize the functional consequences of tumour cell heterogeneity and emergence of clonal dominance in T-ALL. It is well known that human leukaemias and myelomas are oligoclonal at diagnosis; however, relapse is often driven by emergence of an underrepresented clone contained within the primary malignancy^31^,^32^,^33^,^34^,^35^. We have previously created Myc-induced T-ALLs that express a variety of fluorescent proteins and then used cell transplantation to create T-ALLs derived from single leukaemia cells^36^. Using these monoclonal T-ALLs, our experiments sought to dynamically visualize how individual T-ALL clones grow when combined together, testing the hypothesis that inherent functional differences between cells drive the emergence of clonal dominance.

First, we assessed the kinetics of leukaemia regrowth in clones that exhibited wide differences in leukaemia-propagating cell (LPC) frequency and latency. Specifically, we mixed equal numbers of AmCyan, ZsYellow and DsRED-labelled cells isolated from three independent T-ALLs (Supplementary Fig. 2a–c). Following transplantation into the dorsal musculature (3.3 × 10^4^ of each clone, 1.0 × 10^5^ total cells per recipient animal), engrafted fish were imaged by confocal microscopy at 14 and 25 d.p.t. Consistent with our expectations, ZsYellow-labelled T-ALL cells with the highest LPC frequency and fastest growth outcompeted the other clones at each of the time points analysed, making up 48.7% of the leukaemia by 14 d.p.t. and 72.3% by 25 d.p.t. (Supplementary Fig. 2d,e). These data suggest that clonal dominance can result from inherent functional differences between clones.

**Figure 4:**
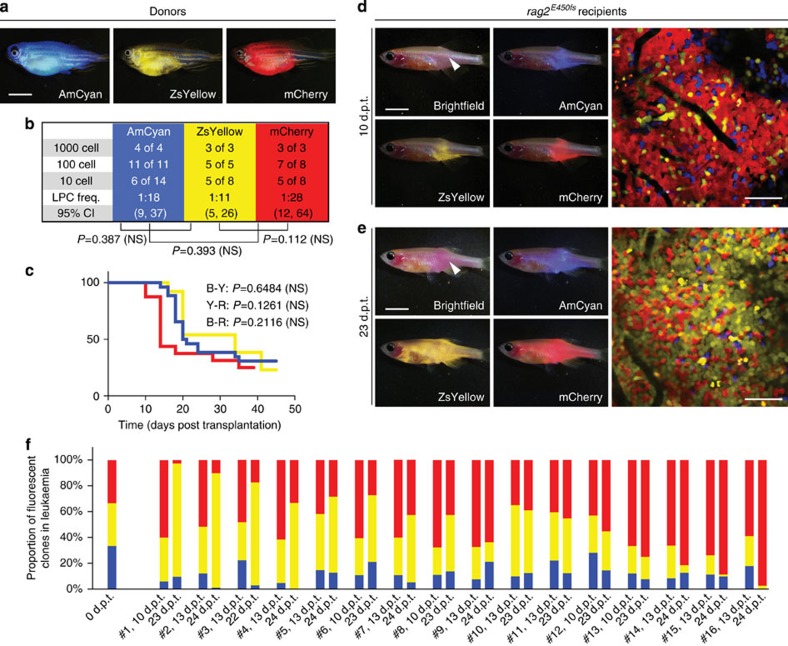
Visualizing the emergence of clonal dominance in Myc-induced T-ALL. (**a**) Donor animals engrafted with monoclonal T-ALL arising in the *CG1* background. (**b**,**c**) Monoclonal T-ALLs were implanted into the syngeneic *CG1* strain fish and assessed for LPC frequency by limiting dilution cell transplantation (**b**) or latency of regrowth (**c**). *P*-values are noted within each panel. Not significant (NS). (**d**,**e**) Confocal imaging of engrafted *rag2*^*E450fs*^ *(casper)* fish at 10 d.p.t. (**d**) and 23 d.p.t. (**e**). White arrow denotes site of injection, and the location for confocal imaging. (**f**) Relative proportions of each fluorescent clone contained within leukaemias from individual engrafted animals (*n*=16 animals). Imaging was completed on the same animals at 10–13 d.p.t. and 22–24 d.p.t. The ZsYellow+ clone dominates leukaemia regrowth in animals #1–3, whereas mCherry+ dominates in animals #13–16. Scale bars equal 5 mm in whole animal images and 50 μm in confocal images.

We next wanted to examine emergence of clonal dominance in T-ALL that had similar LPC frequency and growth kinetics, exploring the notion that clonal dominance may also emerge stochastically *in vivo*. For example, Lgr5+ intestinal crypt stem cells exhibit a pattern of neutral drift, ultimately resulting in regional dominance of a single clone within each villus^37^. In the cancer setting, elegant cell lineage tracing experiments have confirmed that clonal drift can impart regional dominance of Lgr5+ intestinal crypt stem cells in mouse intestinal adenomas^38^. To assess if clonal drift may also account for dominance of T-ALL clones over time, T-ALL clones that had similar growth rates and LPC frequencies were transplanted into recipient *rag2*^*E450fs*^ *(casper)* fish (3.3 × 10^4^ of each clone, 1.0 × 10^5^ total cells per recipient animal). Despite these leukaemias having similar growth rates, latency and LPC frequencies (Fig. 4a–c), the AmCyan+ clone was consistently outcompeted over time, comprising only 9.8±6.4% of the leukaemia by 22–24 d.p.t. (Fig. 4d,e). By contrast, the remaining leukemias contained both mCherry+ and ZsYellow+ cells and in some instances dominance of one clone prevailed (Fig. 4f, *n*=16 animals). To rule out the potential effects of fluorescent labelling may have on the proliferation of leukaemia cells, we repeated the same experiment with another combination of AmCyan, ZsYellow and mCherry T-ALL clones that also shared similar LPC frequency and latency. Interestingly, with this combination, the ZsYellow-labelled cells was reproducibly outcompeted by the AmCyan- or mCherry-labelled cells, with dominance of these latter clones being observed in animals over time (*n*=13 animals total, 2 independent experiments, Supplementary Fig. 3). We conclude that clonal dominance can result from inherent genetic and epigenetic differences between different tumour clones, which was not revealed using traditional limiting dilution cell transplantation approaches that only analyse the growth of individual clonal populations of cells. Moreover, our experiments provided additional evidence that neutral stochastic drift can account for emergence of clonal dominance over time.

### Tumour evolution and metastasis in melanoma

**Figure 5:**
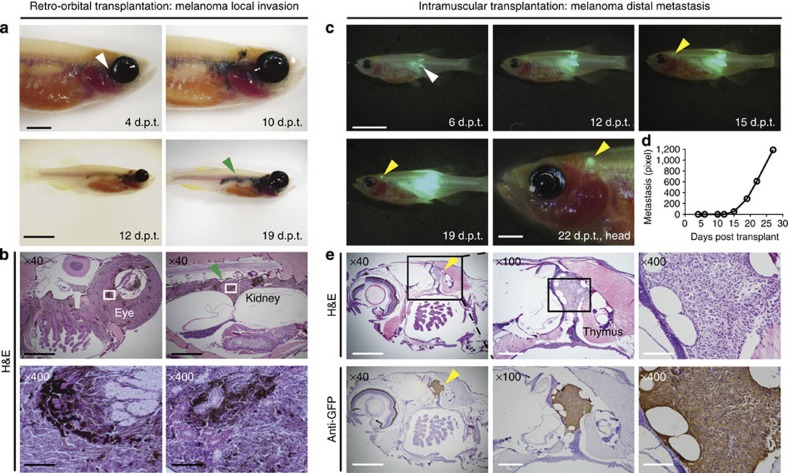
Visualizing melanoma invasion and metastasis following engraftment into *rag2*^*E450fs*^ *(casper)* mutant fish. (**a**) Invasion assays using retro-orbital transplantation of a *BRAF*^*V600E*^*, tp53*^*−/−*^ pigmented melanoma. White arrow denotes the site of injection. Green arrow denotes spread to the kidney marrow that is contiguous with primary tumour growth that has arisen adjacent to the eye. Histological examination confirmed the presence of pigmented melanoma cells at the site of injection (**b**, left panels) and contiguous with the trunk kidney (**b**, right panels). (**c**) Metastasis assays using implantation of non-pigmented, GFP-labelled melanoma cells into the dorsal musculature of *rag2*^*E450fs*^ *(casper)-*recipient fish. White arrow denotes the site of injection. Yellow arrow denotes site of distal metastasis. (**d**) Quantification of metastatic growth as assessed by epi-fluorescence microscopy over time. (**e**) Haematoxylin and eosin-stained sections of the same animal imaged in **c**, confirming metastatic growth of melanoma adjacent to the thymus (top panels) and confirmed by anti-GFP immunostaining on section (bottom panels). Scale bars equal 5 mm for whole animal images, 2 mm in images of heads, 1 mm in × 40 histological images; 300 μm in × 100 histological images and 100 μm in × 400 histological images.

Metastasis is a major clinical challenge for those diagnosed with melanoma and is associated with poor prognosis^39^,^40^. Not all cancer cells can enter the circulation and seed new areas of tumour growth, suggesting that heterogeneity and continued clonal evolution likely drive metastasis in a large subset of cancers^41^. Yet, to date, zebrafish models of *bona fide* metastatic progression have not been fully described. To recapitulate metastasis in adult zebrafish, we first transplanted melanoma cells retro-orbitally into the recipient *rag2*^*E450fs*^ *(casper)* fish. Robust engraftment was observed at the site of injection, followed by local infiltration into structures adjacent the gill, head kidney and kidney marrow (Fig. 5a,b). Yet, with a stringent definition of metastasis, we could not document a single case where metastasis originated from haematogenous spread or was delineated by growth clearly separated from the primary site.

To further refine methods to assess metastatic spread, we next injected GFP-labelled, *BRAF*^*V600E*^*, tp53*^*−/−*^ melanoma cells into the dorsal musculature of *rag2*^*E450fs*^ *(casper)* zebrafish, reasoning that infiltration into muscle would be clearly distinguished from seeding of distant sites within the visceral organs. Using this approach, three out of six primary melanomas metastasized to structures near the thymus and head musculature that were clearly distinct from the primary lesion (Fig. 5c and Supplementary Table 2). In total, 8 of 58 engrafted animals harboured distant metastasis by 30 d.p.t. (Supplementary Table 2). Metastatic growth was easily quantified over time following serial imaging of the same animal (Fig. 5d). GFP+ metastatic lesions were independently confirmed by histo-pathological analysis for morphology, pigmentation and anti-GFP immunostaining on section (Fig. 5e). Using similar approaches, we have also dynamically visualized metastatic tumour growth in neuroblastoma and ERMS (Supplementary Fig. 4), suggesting this approach will likely be broadly useful for assessing the kinetics of metastatic colonization and growth in a wide range of solid tumours.

**Figure 6:**
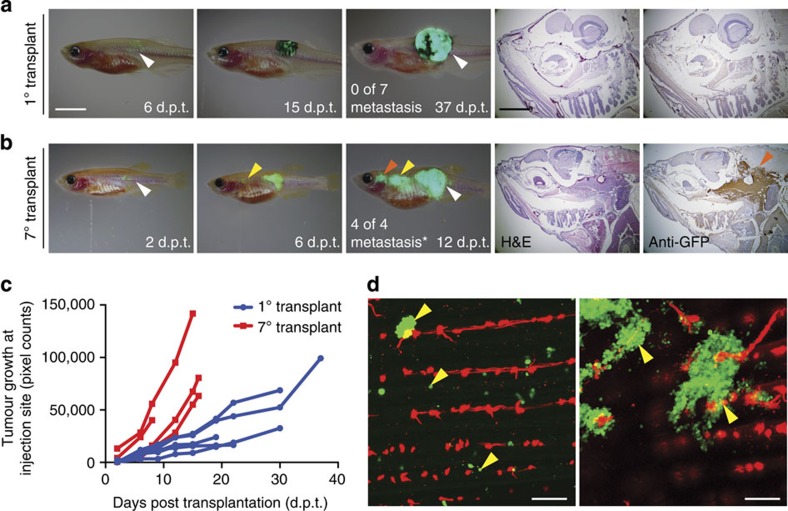
Assessing metastatic potential and the functional consequences of tumour evolution in melanoma. (**a**,**b**) Serial imaging of engrafted GFP-labelled melanoma implanted into adult *rag2*^*E450fs*^ *(capser)* mutant fish. The 1° transplant was pigmented (**a**), whereas the 7° transplanted melanoma had lost pigmentation (**b**). Number of animals with metastatic growth is noted (*P=0.003, Fisher's exact test comparing 1° and 7° transplant). White arrow indicates the site of injection. Yellow and red arrows denote sites of distal metastases. Histological staining (H&E and anti-GFP) of distal metastasis is shown in the right panels. (**c**) Quantification of tumour growth at the site of initial engraftment over time. (**d**) Confocal imaging of micro-metastatic lesions found adjacent to the tail vasculature of *flk1:mCherry; rag2*^*E450fs*^ *(casper)*-recipient fish engrafted with 7° transplant melanoma. Yellow arrows denote micro-metastatic lesions. Scale bars equal 5 mm for whole animal images in **a**,**b**, 2 mm for histology shown in **a**,**b** and 200 μm in **d**.

We next wanted to assess how metastatic potential may change in the same tumour over time and whether serial passaging of individual melanomas could result in phenotypic changes, including increased aggression, invasion and acquisition of metastatic characteristics. Specifically, two non-metastatic *BRAF*^*V600E*^*, p53*^*−/−*^ melanomas at the 1° passage were implanted into the dorsal musculature of *rag2*^*E450fs*^ *(casper)* zebrafish and animals assessed for local engraftment, invasion and metastatic growth following serial passaging. Sectioning and microscopic analysis confirmed that early passaged melanomas were not metastatic and tumours were confined to the site of injection (Fig. 6a and Supplementary Fig. 5a). Following serial passaging, one melanoma continued to resemble the parental tumour and did not change its growth rate or metastatic potential (8 passages, total 191 days *in vivo,* Supplementary Fig. 5b,c). By contrast, the second melanoma lost pigmentation, had significantly accelerated growth and harboured metastatic lesions (7 passages, total 134 days *in vivo,* Fig. 6b,c). With this particular tumour, GFP+ lesions were found to have metastasized to the mesonephric trunk kidney by 6 d.p.t., which eventually spread to the regions adjacent to the thymus and head kidney by 12 d.p.t. (*n*=4 of 4, *P*=0.003, two-tailed Fisher's exact test). Confocal imaging revealed seeding of GFP-labelled cells to distal sites within the tail of the recipient fish, suggesting the formation of micro-metastatic lesions adjacent to *flk1:mCherry*-labelled vasculature (*n*=2, Fig. 6d). Our experiments confirm that not all melanoma cells have metastatic capacity and that evolution of metastatic potential occurs in melanoma, consistent with that have been reported for human melanoma^42^,^43^. In total, our model provides a dynamic imaging platform to visualize the tropism of metastasis.

## Discussion

Imaging cancer cell heterogeneity and the hallmarks of cancer in live animals has been a challenge. Many investigators have developed imaging modalities and complex surgical approaches in rodent models to gain optical access to developing tumours. These approaches often require implantation of imaging windows for repeated high-resolution IVM^10^,^11^. Using these experimental platforms, investigators have successfully imaged several hallmarks of cancer, including migration^44^, intravasation^45^, extravasation^46^, invasion^47^ and metastasis^12^,^14^, as well as responses to chemotherapies *in vivo*^48^. In hope of bypassing the need for surgical intervention and overcoming the limitation of visualizing cancer cells only within predetermined imaging windows, we engrafted fluorescently labelled cancers into optically clear immune-compromised zebrafish and directly imaged tumours using confocal microscopy. Engraftment of fluorescent cancers into the *rag2*^*E450fs*^ *(casper)* zebrafish has facilitated the direct visualization of single cancer cells, functional differences between tumour cell clones and several hallmarks of cancer including acquisition of elevated growth potential, development of new vascular networks and acquired cell motility leading to invasion and metastasis.

The functional consequences of tumour cell heterogeneity have only recently begun to be explored using IVM, and a number of interesting and unexpected tumour cell behaviours have been revealed. For example, our group has utilized fluorescent transgenic approaches to label ERMS cells based on muscle differentiation status^18^. Our experiments uncovered that differentiated ERMS cells seed new areas of growth, followed later by slow-moving, less-differentiated myf5+ TPCs, challenging the notion that cancer stem cells drive invasion and metastasis. Imaging approaches have not been limited to analysis of primary tumours. Rather, Chapman *et al.* have used xenograft cell transplantation of human melanoma cells into larval zebrafish to assess the effects of intra-tumoral heterogeneity on growth^49^. They found that tumours with high invasive capacity could impart migratory potential to poorly invasive cell types. Moreover, the poorly invasive cells could also enhance colonization by aggressive melanomas when co-implanted into larval fish, suggesting cell–cell cross-talk and collaborative signalling networks can enhance migratory and invasive behaviour between melanoma subclones. Using differential labelling of human melanoma cells with GFP and mCherry, these authors were able to dynamically image these processes in live engrafted fish. Here, we performed proof-of-concept experiments to assess the utility of *rag2*^*E450fs*^ *(casper)* zebrafish for visualizing engraftment of heterogeneous ERMS cells in live animals. For example, we directly visualized niche partitioning based on differentiation status using fluorescently labelled ERMS cells. These niches are not anatomically confined by proximity to vessels, in contrast to what has been observed in osteosarcoma^50^ and brain tumours^13^,^51^ where cancer stem cells lie in unique niches adjacent vessels. Furthermore, cell transplantation into orthotopic sites in the *rag2*^*E450fs*^ (*casper)* zebrafish permitted imaging of cells at single-cell resolution in live animals. We envision that similar strategies will likely aid in assessing stromal cell contributions to tumour growth. In total, our cell transplantation and imaging approaches will likely facilitate efforts in defining how niche topology is established and ultimately influences continued tumour growth.

Evidence for continued clonal evolution and selection following treatment has now been seen in a wide range of human cancers. For example, human leukaemias and myelomas are oligoclonal at diagnosis; however, relapse is commonly driven by an underrepresented clone contained within the primary malignancy^31^,^32^,^33^,^34^,^35^. Similar dynamics of heterogeneity has also been observed in solid tumours. For example, triple-negative breast cancers are comprised of heterogeneous clones that harbour a wide spectrum of mutations that can change from the time of diagnosis to relapse^52^,^53^. Altering frequencies of specific clones have also been observed during tumour progression in pancreatic cancer^54^,^55^ and brain tumours^56^. Elegant cell lineage tracing experiments in mice have shown experimentally that stochastic clonal drift can contribute to regional dominance of Lgr5+ intestinal crypt stem cells in mouse intestinal adenomas^38^; yet it is unclear if this pattern applies to a wider array of cancer subtypes. Here, we have experimentally recreated tumour cell heterogeneity by implanting equal numbers of fluorescently labelled T-ALL clones into the *rag2*^*E450fs*^ *(casper)* zebrafish. By competing these T-ALL clones together, we find that one clone was consistently outcompeted over time despite having equal self-renewal potential and overall growth kinetics. Our data suggest that subtle functional variations can exist within clones that may not be uncovered using traditional cell transplantation assays that assess tumour regrowth of only single clones, likely underestimating differences in latency and LPC frequency. Alternatively, these data may suggest that clones actively suppress the growth of other clones and/or secrete factors to enhance growth of only related cells. This interpretation is consistent with recent findings in breast cancer where paracrine factor signalling from one clone can alter growth of unrelated clones within the tumour^57^. Taken together, these *in vivo* competition experiments have uncovered interesting and potentially new biology that could not have been discovered using traditional limiting dilution cell transplantation approaches, facilitating the dynamic emergence of clonal dominance within the same animal overtime.

Metastasis can be experimentally assessed in mouse models by engrafting tumour cells into the vasculature and then identifying metastatic lesions at necropsy. Alternatively, luciferase bioluminescent imaging and non-invasive, whole-body imaging methods have also been developed, including computed X-Ray tomography, positron emission tomography and magnetic resonance imaging. These imaging modalities are limited to detection of tumours that are 200 μm in diameter, expensive and largely inaccessible to many laboratories^58^,^59^,^60^. Here, we have optimized cell transplantation into adult, immune-deficient *rag2*^*E450fs*^ *(casper)* zebrafish in order to dynamically visualize tumour cell migration, invasion and metastasis at high resolution. We performed cell transplantation of fluorescently labelled zebrafish tumour into the optic vessels and dorsal musculature, establishing that metastatic progression is best assessed following injection into the dorsal musculature. Furthermore, we have been able to visualize the dissemination of micro-metastatic lesions to sites adjacent to the tail vasculature in *flk1:mCherry; rag2*^*E450fs*^ *(casper)* zebrafish using simple confocal imaging. Finally, using serial transplantation, we were able to evolve melanomas with high metastatic potential, providing a novel platform for identifying driver mutations that are specifically correlated with progression. We envision that facile genetic approaches including transgenesis and genetic knock-out using CRISPR/Cas9 will quickly make zebrafish the choice experimental model for assessing gene pathways that modulate tumour progression and metastasis, especially in the transplantation setting.

## Methods

### Creation of *rag2* ^*E450fs*^ *(casper)* homozygous mutant zebrafish

Zebrafish studies were approved by the Massachusetts General Hospital Subcommittee on Research Animal Care (protocol #2011N000127).

*rag2*^*E450fs*^ *(casper)* homozygous mutant zebrafish were created by crossing *rag2*^*E450fs*^ *(rag2*^*fb101*^) mutant fish into the *casper* background^19^. Animals were maintained as *rag2*^*E450fs/+*^*; roy*^*−/−*^*; mitfa*^*−/−*^ lines and in-crossed to generate triple mutant animals. Resulting progeny were fin clipped and genotyped using the same method as we previously published^22^. Specifically, genomic DNA was extracted using the Hotshot method described by Meeker *et al.*^61^, and subjected to PCR using forward primer 5′-ACTGCTCTAGTTGCAATTCCT-3′ and reverse primer 5′-AGCTGGGGTCATCTTCAGT-3′. PCR was completed using 94 °C denaturation for 30 s, 54 °C annealing for 30 s and 68 °C elongation for 45 s (35 cycles). PCR-amplified products were then incubated at 37 °C overnight with XcmI, which created a single cut in the mutant allele. Finally, DNA products were resolved either on a 2% agarose gel or using the Qiaxcel genotyping system. For neovascularization studies, *flk1:mCherry* transgenic fish^62^ were crossed with *rag2*^*E450fs*^ *(casper)* fish and in-crossed to create compound mutant animals.

### Tumour creation and cell transplantation into zebrafish

Primary and serially passaged tumours were derived from established transgenic zebrafish models. For example, T-ALLs were created by co-microinjection of linearized *rag2:cMyc* and fluorescent transgenic reporters into one-cell stage *CG1* fish^36^,^63^ (Figs 1 and 4 and Supplementary Figs 1–3). GFP-labelled ERMS were created by co-microinjection of linearized *rag2:kRAS*^*G12D*^ and *rag2:GFP* transgenes into one-cell stage *CG1* fish^64^ (Fig. 2); double-fluorescent ERMS were created by microinjection of linearized *rag2:kRAS*^*G12D*^ into one-cell-stage stable transgenic *myf5:GFP; mylpfa:mCherry CG1* fish^18^ (Fig. 1 and Supplementary Fig. 4). Triple-fluorescent transgenic ERMSs were created by microinjecting the linearized *rag2:kRAS*^*G12D*^ transgene into one-cell stage stable transgenic *myf5:GFP; myogenin-H2b:mRFP; mylpfa:lyn-cyan* zebrafish in the AB background (Fig. 3). Melanomas were created by overexpression of MiniCoopR-EGFP in the embryos of *Tg (mitfa:BRAF*^*V600E*^*); mitfa*^*−/−*^*; tp53*^*−/−*^ or *Tg (mitfa:BRAF*^*V600E*^*); mitfa*^*−/−*^*; tp53*^*−/−*^; *alb*^*−/−*^ zebrafish^27^ (Figs 1, 2, 5 and 6 and Supplementary Fig. 5), and were a kind gift from the Houvras laboratory. The official ZFIN designation for the *tp53*^*−/−*^ zebrafish line is *zdf1*^*M214K*^, originally reported by Berghmans *et al.*^65^. Neuroblastomas were a kind gift from the Look laboratory^24^.

Cell transplantation experiments utilized both male and female *rag2*^*E450fs*^ *(casper)* homozygous mutant zebrafish. Recipient animals were transplanted at 2–4 months of age. Tumour cell transplantations were completed by intra-peritoneal, intra-muscular and retro-orbital injections^66^,^67^,^68^. Intra-muscular and retro-orbital injections were completed using microinjection of 2–3 μl of cell suspension. Recipient fish were scored for tumour engraftment, growth, invasion or metastasis by epi-fluorescent microscopy every 3–4 days until 30 d.p.t. or when animals were moribund.

### Confocal imaging of neovascularization and micro-metastasis

*rag2*^*E450fs*^ *(casper)* animals were engrafted with GFP-labelled ERMS or melanoma. To visualize tumour vasculature, animals were injected with Quantum dot 655 reagent (Qtracker 655, 405–615 nm excitation, 655 nm emission, 2.0 μM, Life Technologies Cat# Q21021MP). Specifically, animals were injected with freshly prepared 1:3 diluted reagent (1 volume Qtracker 655 solution diluted in 2 volumes of 0.9x PBS, yielding a final concentration of 0.66 μM) using a 26s gauge Hamilton syringe (4 μl injected intra-peritoneally, and 2 μl directly into the dorsal aorta). After 30 min, animals were anaesthetized using 168 mg l^−1^ Tricaine (MS-222, pH=7.5) and placed onto a 12.0-mm glass bottom imaging plate (Thermo Scientific, Cat# 150680). Under anaesthesia, motor functions of the animal were greatly reduced and operculum movements were significantly slowed. Each animal was imaged under anaesthesia for 1–2 min, allowing enough time for z-stack imaging or multi-position single plane imaging using confocal microscopy. To ensure optimal survival of the adult zebrafish being imaged, anaesthetized animals were simply laid down on the side in the imaging dish, with the tumour facing towards the lens. No agarose embedding of the sample was used. Imaging was completed using an inverted Zeiss LSM 710 confocal microscope. For neovascularization (Fig. 2) and micro-metastasis imaging (Fig. 6d), z-stack imaging at × 100 magnification was achieved with a × 10 objective (numerical aperture=0.45, coverglass thickness=0.17 mm, working distance=2.0 mm). Image series were acquired in the ZEN software (single pass point scanning at ∼10 μm per step over 100–200 μm distances). Fluorescent imaging was completed using the following settings: GFP: excitation=488 nm, emission=503–528 nm; mCherry/mRFP: excitation=561 nm, emission=602–624 nm; Qtracker 655: use AlexaFlour647 setting, excitation=633 nm, emission=638–755 nm. Laser intensity percentages were set to be between 10 and 20% depending on the brightness of the fluorescent labels used. The ‘Best Signal' option in the ‘Smart Setup' was used for easy modulation of the gating of each colour. For imaging fluorescent combinations, gate settings were manually adjusted to avoid overlap of fluorescent signals. For z-stack images, planes were merged based on maximum intensity in Fiji (ImageJ). After imaging, fish were immediately transferred into recovery tanks that contained fresh system water, and later returned to the facility.

### Confocal imaging of heterogeneity in ERMS and T-ALL

Multi-colour confocal fluorescence imaging was completed in both ERMS and T-ALL. Specifically, in ERMS (Fig. 3) and T-ALL (Fig. 4 and Supplementary Figs 2,3), we simultaneously imaged using three-colour combinations including AmCyan (excitation=458 nm, emission=472–508 nm), GFP (excitation=488 nm, emission=493–586 nm), ZsYellow (excitation=514 nm, emission=521–547 nm), DsRED (excitation=561 nm, emission=575–703 nm) and mCherry/mRFP (excitation=561 nm, emission=602–623 nm). Laser intensity percentages were set to be 18%. For imaging at single-cell resolution, × 400 magnification was achieved with a × 40 water emersion objective (NA=1.3, coverglass thickness=0.14–0.19 mm, working distance=0.62 mm for 0.17 mm coverglass). The ‘Best Signal' option in the ‘Smart Setup' was used for easy modulation of the gating of each colour. For T-ALL, proportions of each clone were quantified in the Fiji (ImageJ) software by measure of area covered by each fluorescent colour (Fig. 4 and Supplementary Fig. 2,3f), or manual cell counts (Supplementary Fig. 3g). Both quantification methods produce consistently similar results.

### Histological examination

Tumour histology was performed on animals as previously described^22^. Briefly, animals were fixed in 4% paraformaldehyde, embedded in paraffin and step sectioned. Adjacent slides were stained with haematoxylin and eosin or anti-GFP (Living Colors Monoclonal Antibody JL-8, Clontech, 1:1,000 dilution).

### Statistical methods

Leukaemia propagating cell frequencies were calculated using the extreme limiting dilution analysis software (http://bioinf.wehi.edu.au/software/elda/). Differences in the LPC frequency were reported with a 95% confidence interval. Differences of latency were assessed by log-rank (Mantel-Cox) tests (Fig. 4 and Supplementary Figs 2,3). In the melanoma evolution experiments (Fig. 6), increased metastatic potential was assessed by Fisher's exact test, comparing the number of animals with metastatic disease at 1° transplant with those found after 7° transplant.

## Additional information

**How to cite this article:** Tang, Q. *et al.* Imaging tumour cell heterogeneity following cell transplantation into optically clear immune-deficient zebrafish. *Nat. Commun.* 7:10358 doi: 10.1038/ncomms10358 (2016).

## Supplementary Material

Supplementary Information Supplementary Figures 1-5 and Supplementary Tables 1-2.
