# Supplementary material for: Imaging tumour cell heterogeneity following cell transplantation into optically clear immune-deficient zebrafish
Source: Nat Commun. 2016 Jan 21;7:10358. doi: 10.1038/ncomms10358 (PMC4735845; doi:10.1038/ncomms10358)
Supplement: Supplementary Information — Supplementary Figures 1-5 and Supplementary Tables 1-2. [file ncomms10358-s1.pdf]

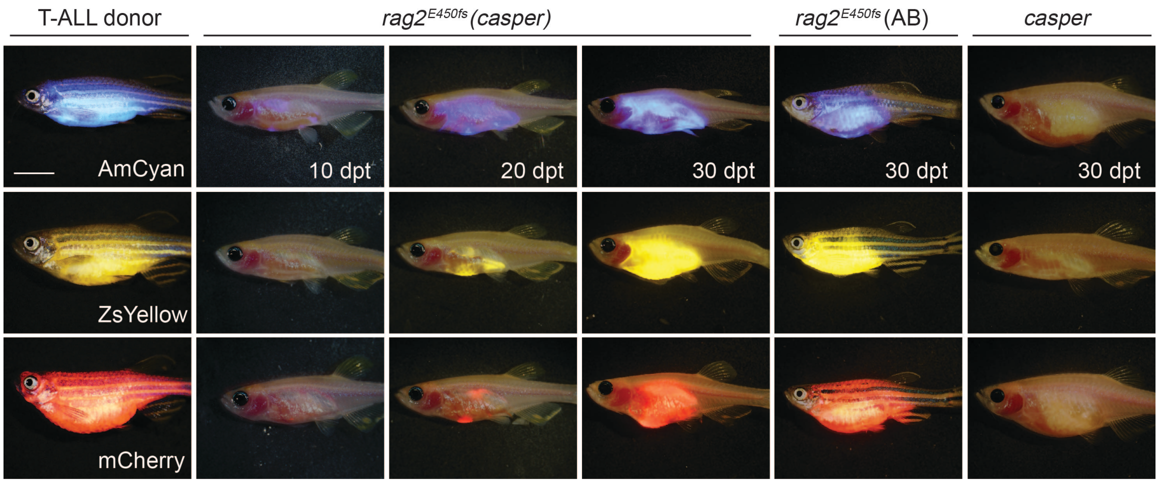

Supplementary Figure 1: Imaging T-ALL progression and growth in transplanted *rag2*$^{\boldsymbol{E450fs}}$ *(casper)* fish. Monoclonal T-ALLs were serially passaged in *CG1* strain fish and then used as donors (left panel). Cells were transplanted intra-peritoneally into *rag2*^{E450fs} *(casper), rag2*^{E450fs} (AB), and unconditioned, *casper*-strain recipient fish (1.0×10^5 cells per recipient animal). Merged brightfield and fluorescent images are shown at 10, 20 and 30 dpt. Scale bars equal 5 mm.

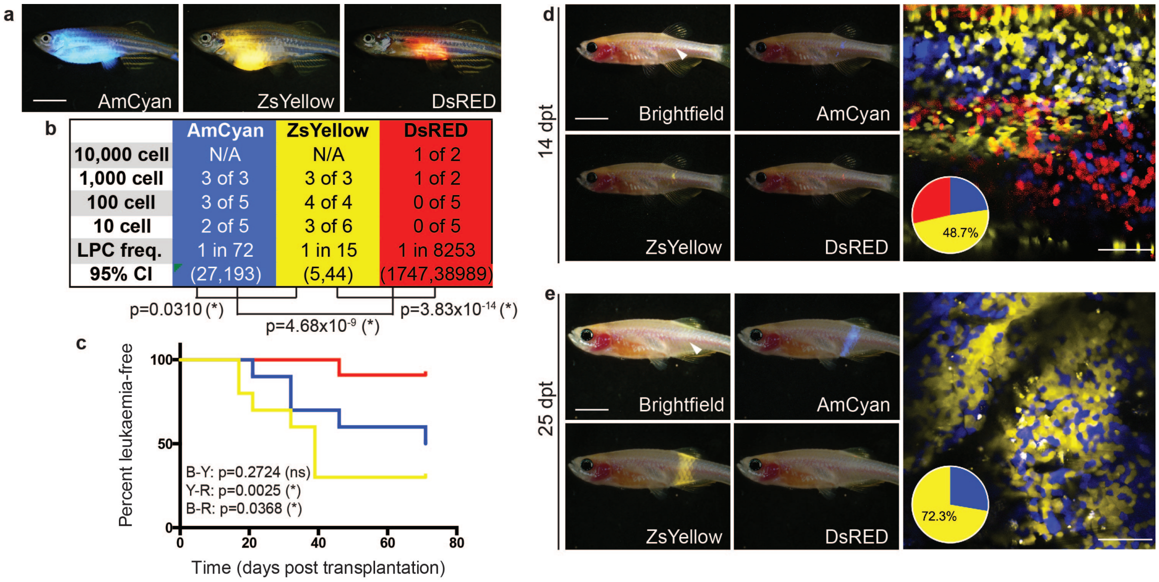

Supplementary Figure 2: Emergence of clonal dominance can result from inherent functional differences between T-ALL clones. (**a**) Donor animals engrafted with monoclonal T-ALL arising in the *CG1* background. (**b-c**) Monoclonal T-ALLs were implanted into syngeneic *CG1* strain fish and assessed for LPC frequency by limiting dilution cell transplantation (**b**) or latency of regrowth (**c**). T-ALLs have significantly different LPC frequency and latency, with p-values noted on each panel. (**d-e**) Confocal imaging of engrafted $rag2^{E450fs}$ *(casper)* fish at 14 dpt (**d**) and 25 dpt (**e**). White arrow denotes site of injection and imaging. Pie chart shows the relative proportion of each fluorescent clone contained within the imaging panel shown. Scale bars equal 5 mm in whole animal images and 50 μm in confocal images.

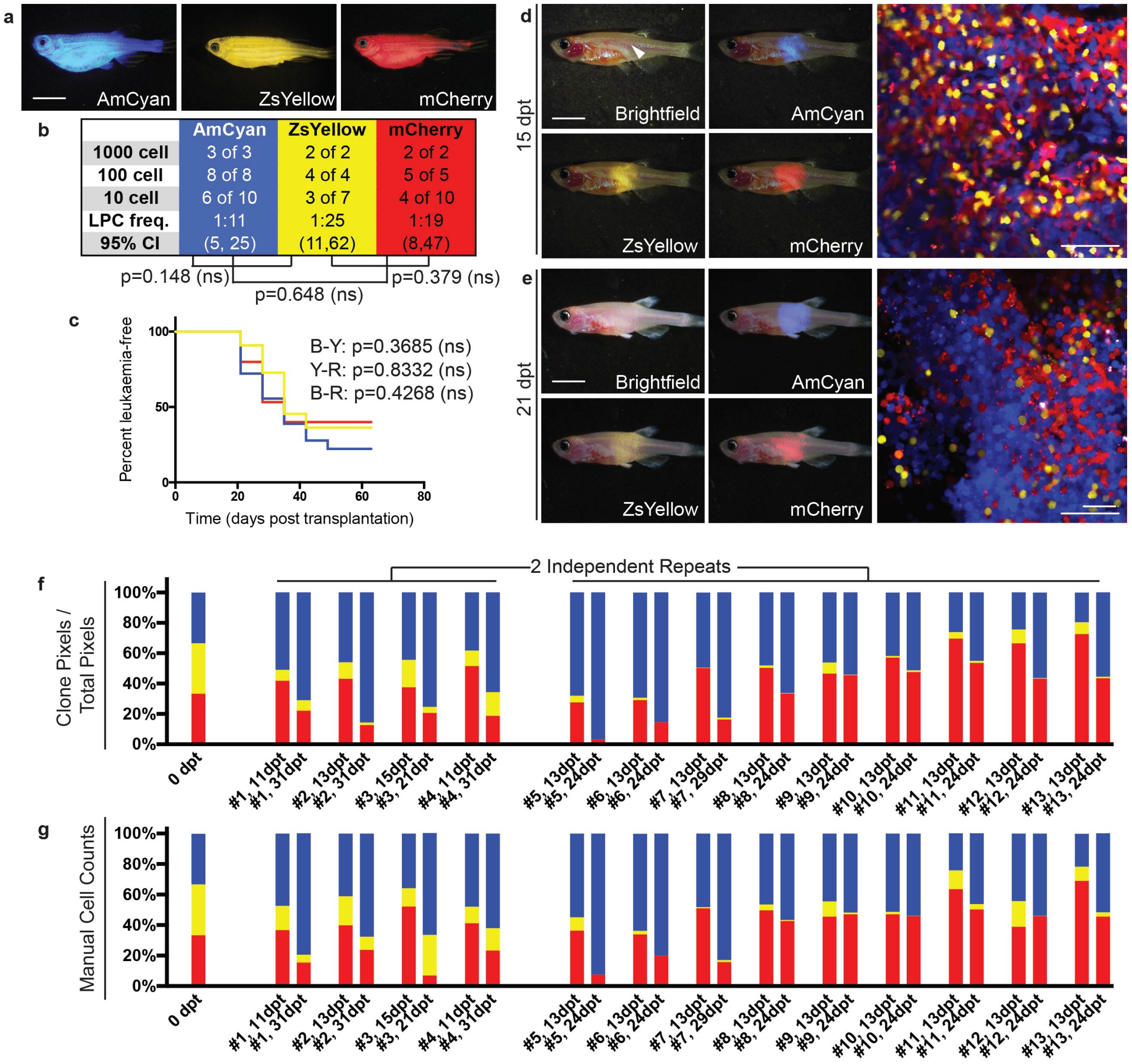

Supplementary Figure 3: Emergence of clonal dominance among three similar T-ALL clones. (**a**) Donor animals engrafted with monoclonal T-ALL arising in the *CG1* background. (**b-c**) Monoclonal T-ALLs were implanted into syngeneic *CG1* strain fish and assessed for LPC frequency by limiting dilution cell transplantation (**b**) or latency of regrowth (**c**). T-ALLs have similar LPC frequency and latency, with p-values noted on each panel. (**d-e**) Confocal imaging of engrafted *rag2*^{E450fs} *(casper)* fish at 15 dpt (**d**) and 21 dpt (**e**). White arrow denotes site of injection and imaging. (**f-g**) Relative proportions of each fluorescent clone contained within individual engrafted animals quantified by

fluorescence area within confocal images (**f**) and manual cell counts (**g**). Scale bars equal 5 mm in whole animal images and 50 μm in confocal images.

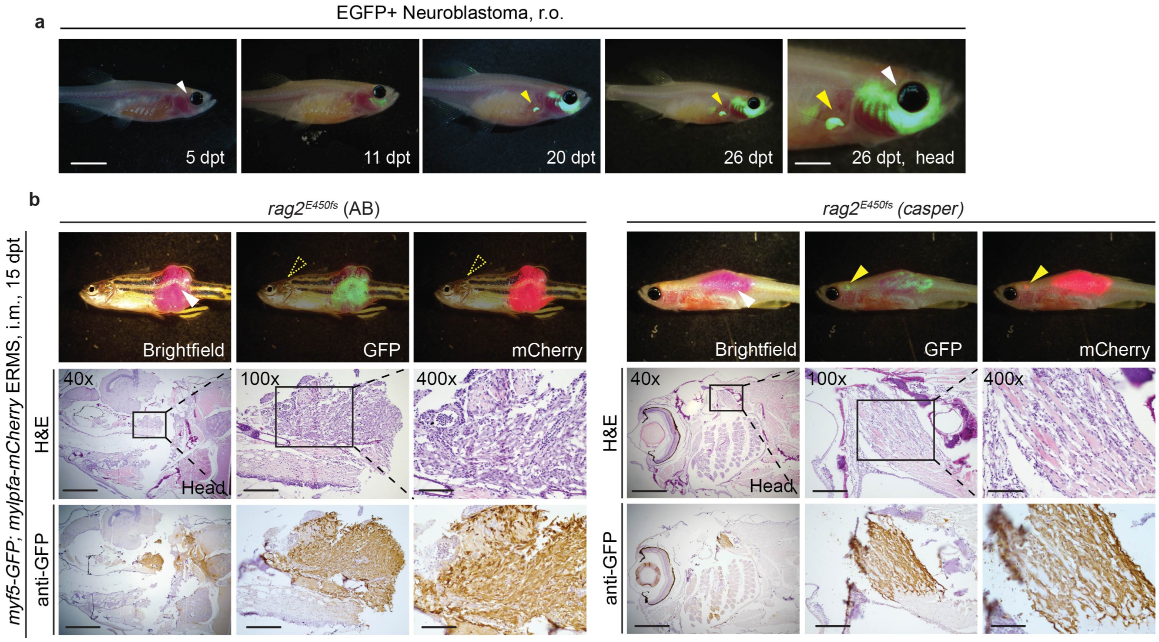

Supplementary Figure 4: Visualising neuroblastoma and ERMS metastasis in transplanted *rag2*^{E450fs} *(casper)* fish. (**a**) Retro-orbital transplantation of a GFP-labelled neuroblastoma. White arrow indicates the site of injection; yellow arrow indicates metastasis to a region near the liver. (**b**) Intra-muscular transplantation of ERMS into *rag2*^{E450fs} recipient fish. White arrow indicates the site of injection; yellow arrows indicate the site of metastasis. Note that metastasis can be directly visualised by epi-fluorescence microscopy in the *rag2*^{E450fs} *(casper)* recipients (yellow arrow) but not the *rag2*^{E450fs} (AB) (open yellow arrow). H&E and anti-GFP staining on sections of the recipient animals confirmed the location of metastasis. Scale bars equal 5 mm for whole animal images, 2 mm in images of heads, 1 mm in 40x histological images**;** 300 μm in 100x histological images; and 100 μm in 400x histological images.

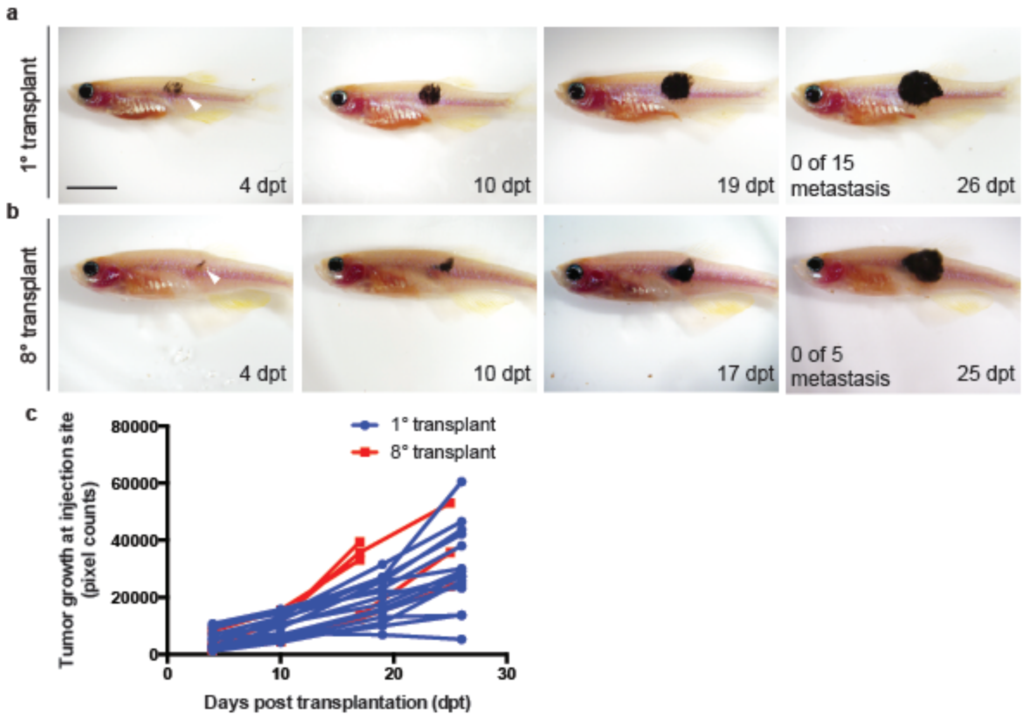

Supplementary Figure 5: Serial passaging did not result in increased metastatic potential or growth in one melanoma. (**a-b**) Serial imaging of engraftment of a pigmented melanoma into *rag2*^{E450fs} *(casper)* recipient fish. 1° transplant (**a**) and 8° transplant (**b**). White arrow indicates the site of injection. (**c**) Quantification of tumour growth within individual engrafted fish. Scale bar equals 5 mm.

Supplementary Table 1: Transplantations performed in this study

Donor					Recipient	Age of recipients (months)	Method of transplantation	Number of tumour cells transplanted per recipient	Recipients engrafted	Total trans-planted
**Tumour type**	**Transgenics**	**Back-ground**	**Fluorescent marker**	**Donor animal**						
T-cell lymphoblastic leukaemia (T-ALL)	*Tg (rag2:cMyc)*	*CG1*	AmCyan	Monoclonal in *CG1* (Supplementary Fig. 1)	*rag2*^{E450fs} *(casper)*	4	intra-peritoneal (i.p.)	1.0x10^{5} cells in 5 µl	5	6
					Wild-type (casper)	4			0	6
					rag2^{E450fs} *(AB)*	4			6	6
			ZsYellow	Monoclonal in *CG1* (Fig. 1a; Supplementary Fig. 1)	*rag2*^{E450fs} *(casper)*	4			3	5
					Wild-type (casper)	4			0	10
					rag2^{E450fs} *(AB)*	4			6	6
			mCherry	Monoclonal in *CG1* (Supplementary Fig. 1)	*rag2*^{E450fs} *(casper)*	4			3	5
					Wild-type (casper)	4			0	6
					rag2^{E450fs} *(AB)*	4			4	5
			AmCyan + ZsYellow + mCherry	Monoclonal in *CG1* (Fig. 4)	*rag2*^{E450fs} *(casper)*	2.5	intra-muscular (i.m.)	3.3x10^{4} of each clone, 1.0x10^{5} cells in total per recipient animal, in 2 µl	17	17
			AmCyan + ZsYellow + DsRED	Monoclonal in *CG1* (Supplementary Fig. 2)	*rag2*^{E450fs} *(casper)*	2			3	16
			AmCyan + ZsYellow + mCherry	Monoclonal in *CG1* (Supplementary Fig. 3)	*rag2*^{E450fs} *(casper)*	3.5			9	10
					rag2^{E450fs} *(casper)* (repeat)	2			10	12
Neuro-blastoma	*Tg(dβh:EGFP-MYCN; dβh:ALK*^{F1174L}*)*	AB	EGFP	primary	*rag2*^{E450fs} *(AB)*	3	intra-peritoneal (i.p.)	5.0x10^{5} cells in 5 µl	7	7
				1° transplant in *rag2*^{E450fs} (AB)	*rag2*^{E450fs} *(AB)*	3	intra-peritoneal (i.p.)	1.0x10^{6} cells in 5 µl	4	4
				2° transplant in *rag2*^{E450fs} (AB) (Fig. 1b)	*rag2*^{E450fs} *(AB)*	3.25	intra-peritoneal (i.p.)	1.0x10^{6} cells in 5 µl	3	3
					rag2^{E450fs} *(casper)*	3.75	intra-peritoneal (i.p.)	5.0x10^{5} cells in 2.5 µl	5	6
					rag2^{E450fs} *(casper)*	3.75	retro-orbital (r.o.) (Supplementary Fig. 4a)	4.0x10^{5} cells in 2 µl	14	17
					Wild-type (casper)	3.75	retro-orbital (r.o.)	4.0x10^{5} cells in 2 µl	0	15

Embryonal rhabdomyo-sarcoma (ERMS)	*Tg (rag2:kRAS*^{G12D}*; ICN)*	*CG1*	*myf5:GFP; mylpfa:mCherry*	3° transplant in *CG1*	*rag2*^{E450fs} *(casper)*	4	intra-peritoneal (i.p.)	5.0x10^5 cells in 5 µl	5	6
					Wild-type (casper)	4			0	7
					rag2^{E450fs} *(AB)*	3			2	2
					rag2^{E450fs} *(casper)*	4	intra-muscular (i.m.) (Fig. 1c; Supplementary Fig. 4b)	3.0x10^5 cells in 3 µl	13	13
					Wild-type (casper)	4			0	9
					rag2^{E450fs} *(AB)*	3			6	6
		CG1	*myf5:GFP; mylpfa:mCherry*	4° transplant in *CG1*	*rag2*^{E450fs} *(AB)*	3	intra-muscular (i.m.)	3.3x10^5 cells in 2 µl	4	11
					rag2^{E450fs} *(casper)*	3	intra-muscular (i.m.)		9	14
					rag2^{E450fs} *(casper)*	3	retro-orbital (r.o.)		14	14
	Tg (rag2:kRAS^{G12D}*)*	AB	*myf5:GFP; myogenin-H2b:mRFP; mylpfa:lyn-cyan*	primary	*flk1:mCherry; rag2*^{E450fs} *(casper)*	2	intra-peritoneal (i.p.)	2.0x10^4 cells in 5 µl	2	2
				1° transplant in *flk1:mCherry; rag2*^{E450fs} *(casper)* (Fig. 3)	*flk1:mCherry; rag2*^{E450fs} *(casper)*	3	intra-muscular (i.m.)	4.0x10^5 cells in 2 µl	4	4
					rag2^{E450fs} *(casper)*	3	intra-muscular (i.m.)		3	3
		CG1, (tp53$^{+/-}$*)*^{2}	*rag2:GFP*	1° transplant in *CG1*	*flk1:mCherry; rag2*^{E450fs} *(casper)*	4	intra-muscular (i.m.)	8.0x10^4 cells in 2 µl	4	4
					rag2^{E450fs} *(casper)*	2.5	intra-muscular (i.m.)		5	5
		CG1	*rag2:GFP*	primary	*flk1:mCherry; rag2*^{E450fs} *(casper)*	2.5	intra-muscular (i.m.)	1.6x10^5 cells in 2 µl	5	5
					rag2^{E450fs} *(casper)*	2.5	intra-muscular (i.m.)		5	5
		CG1	*rag2:GFP*	primary	*flk1:mCherry; rag2*^{E450fs} *(casper)*	2.5	intra-muscular (i.m.)	2.0x10^5 cells in 2 µl	5	5
					rag2^{E450fs} *(casper)*	2.5	intra-muscular (i.m.)		5	5
		CG1	*rag2:GFP*	2° transplant in *CG1*	*flk1:mCherry; rag2*^{E450fs} *(casper)* (Fig. 2c,d)	4	intra-muscular (i.m.)	5.0x10^5 cells in 2 µl	5	5
					rag2^{E450fs} *(casper)*	4	intra-muscular (i.m.)		5	5
Melanoma	*Tg (mitfa:BRAF*^{V600E}*); tp53*$^{-/-}$*; mitfa*$^{-/-}$	*nacre*	*Tg (MiniCoopR:GFP)*	primary	*rag2*^{E450fs} *(casper)*	3	intra-peritoneal (i.p.)	1.5x10^5 cells in 5 µl	6	6
				1° transplant in *rag2*^{E450fs}*(casper)*	*flk1:mCherry; rag2*^{E450fs} *(casper)*	3.5	intra-muscular (i.m.)	5.0x10^5 cells in 2 µl	8	8
	Tg (mitfa:BRAF^{V600E}*); tp53*$^{-/-}$*; mitfa*$^{-/-}$	*nacre*	*Tg (MiniCoopR:GFP)*	primary	*rag2*^{E450fs} *(casper)*	3	intra-peritoneal (i.p.)	8.0x10^5 cells in 5 µl	3	3
				1° transplant in *rag2*^{E450fs}*(casper)*	*flk1:mCherry; rag2*^{E450fs} *(casper)*	3	intra-muscular (i.m.)	5.0x10^5 cells in 2 µl	7	8
	Tg (mitfa:BRAF^{V600E}*); tp53*$^{-/-}$*; mitfa*$^{-/-}$	*nacre*	*Tg (MiniCoopR:GFP)*	primary	*rag2*^{E450fs} *(casper)*	3	intra-peritoneal (i.p.)	1.8x10^6 cells in 5 µl	2	2
				1° transplant in *rag2*^{E450fs}*(casper)*	*flk1:mCherry; rag2*^{E450fs} *(casper)*	3	intra-muscular (i.m.)	5.0x10^5 cells in 2 µl	7	8

	Tg (mitfa:BRAF^{V600E}*); tp53*$^{-/-}$*; mitfa*$^{-/-}$*; alb*$^{-/-}$	*nacre*	*Tg (MiniCoopR:GFP)*	primary	*rag2*^{E450fs} *(casper)*	4	intra-peritoneal (i.p.)	5.5x10^5 cells in 5 µl	3	3
					rag2^{E450fs} *(AB)*	1.5	intra-peritoneal (i.p.)		3	3
				1° transplant in *rag2*^{E450fs}*(casper)*	*rag2*^{E450fs} *(casper)*	3	intra-muscular (i.m.) (Fig. 5c-e)	1.0x10^5 cells in 2 µl	14	14
						3	retro-orbital (r.o.)		12	15
				4° transplant in *rag2*^{E450fs}*(casper)*	*rag2*^{E450fs} *(casper)*	3	intra-muscular (i.m.)	5.0x10^5 cells in 3 µl	3	3
				6° transplant in *rag2*^{E450fs}*(casper)*	*flk1:mCherry; rag2*^{E450fs} *(casper)* (Fig. 2b)	2.5	intra-muscular (i.m.)	5.0x10^5 cells in 2 µl	5	5
					rag2^{E450fs} *(casper)* (Fig. 2a)	3	intra-muscular (i.m.)	1.0x10^5 cells in 2 µl	4	5
				7° transplant in *rag2*^{E450fs}*(casper)*	*flk1:mCherry; rag2*^{E450fs} *(casper)*	2	intra-muscular (i.m.)	5.0x10^5 cells in 2 µl	5	5
					rag2^{E450fs} *(casper)*	2	intra-muscular (i.m.)		5	5
	Tg (mitfa:BRAF^{V600E}*); tp53*$^{-/-}$*; mitfa*$^{-/-}$	*nacre*	*Tg (MiniCoopR:GFP)*	primary	*rag2*^{E450fs} *(casper)*	4	intra-peritoneal (i.p.)	3.5x10^5 cells in 5 µl	5	5
				1° transplant in *rag2*^{E450fs}*(casper)*	*rag2*^{E450fs} *(casper)*	4	intra-muscular (i.m.) (Fig. 6a)	5.0x10^5 cells in 2 µl	7	7
						4	retro-orbital (r.o.)		15	17
				5° transplant in *rag2*^{E450fs}*(casper)*	*rag2*^{E450fs} *(casper)*	2.5	intra-muscular (i.m.)	5.0x10^5 cells in 2 µl	4	4
				7° transplant in *rag2*^{E450fs}*(casper)*	*rag2*^{E450fs} *(casper)*	3	intra-muscular (i.m.) (Fig. 6b,d)	5.0x10^5 cells in 2 µl	4	4
	Tg (mitfa:BRAF^{V600E}*); tp53*$^{-/-}$*; mitfa*$^{-/-}$	*nacre*	*Tg (MiniCoopR:GFP)*	Primary (Fig. 1d)	*rag2*^{E450fs} *(casper)*	4	intra-peritoneal (i.p.)	4.5x10^5 cells in 5 µl	5	5
				1° transplant in *rag2*^{E450fs}*(casper)*	*rag2*^{E450fs} *(casper)*	4	intra-muscular (i.m.) (Supplementary Fig. 5a)	3.5x10^5 cells in 2 µl	15	15
						4	retro-orbital (r.o.) (Fig. 5a)		13	15
				4° transplant in *rag2*^{E450fs}*(casper)*	*rag2*^{E450fs} *(casper)*	3	intra-muscular (i.m.)	5.0x10^5 cells in 3 µl	3	3
				8° transplant in *rag2*^{E450fs}*(casper)*	*flk1:mCherry; rag2*^{E450fs} *(casper)*	4	intra-muscular (i.m.) (Supplementary Fig. 5b)	5.0x10^5 cells in 2 µl	5	5

Supplementary Table 2: Metastasis assessments in this study

Donor					Recipients	Method of Transpl-antation	Dosage per recipient (cells)	Engraftment and Metastasis			Total engraft-ed	Total trans-planted
**Tumour type**	**Transgenics**	**Back-ground**	**Fluorescence marker**	**Donor animal**				**Local engraft-ment only**	**Invasion**	**Distal Metastasis**		
Neuro-blastoma	*Tg(dβh:EGFP-MYCN; dβh:ALK^{F1174L})*	AB	*dβh:EGFP*	2° transplant in *rag2^{E450fs}* (AB)	*rag2^{E450fs} (casper)*	r.o.	$4.0x10^5$	13	0	1 (near liver) (Supplementary Fig. 4a)	14	17
ERMS	*Tg (rag2:kRAS^{G12D};ICN)*	*CG1*	*myf5:GFP; mylpfa:mCherry*	3° transplant in *CG1*	*rag2^{E450fs} (AB)*	i.m.	$3.0x10^5$	5	0	1 (muscle tissue adjacent to thymus) (Supplementary Fig. 4b)	6	6
					rag2^{E450fs} (casper)	i.m.		12	0	1 (muscle tissue adjacent to thymus) (Supplementary Fig. 4b)	13	13
	Tg (rag2:kRAS^{G12D};ICN)	*CG1*	*myf5:GFP; mylpfa:mCherry*	4° transplant in *CG1*	*rag2^{E450fs} (AB)*	i.m.	$3.3x10^5$	4	0	0	4	11
					rag2^{E450fs} (casper)	i.m.		8	1 (trunk kidney)	0	9	14
					rag2^{E450fs} (casper)	r.o.		8	5 (invasion into cranial cavity, no kidney involvement)	1 (head kidney)	14	14
	Tg (rag2:kRAS^{G12D})	*CG1*	*rag2:GFP*	2° transplant in *CG1*	*flk1:mCherry; rag2^{E450fs} (casper)*	i.m.	$5.0x10^5$	4	1 (whole kidney)	0	5	5
					rag2^{E450fs} (casper)	i.m.	$5.0x10^5$	5	0	0	5	5
Melanoma	*Tg(mitfa:BRAF^{V600E}); tp53$^{-/-}$; mitfa$^{-/-}$*	*nacre*	*MiniCoopR:GFP*	1° transplant in *rag2^{E450fs} (casper)*	*flk1:mCherry; rag2^{E450fs} (casper)*	i.m.	$5.0x10^5$	7	0	0	7	8
	Tg(mitfa:BRAF^{V600E}); tp53$^{-/-}$; mitfa$^{-/-}$	*nacre*	*MiniCoopR:GFP*	1° transplant in *rag2^{E450fs} (casper)*	*flk1:mCherry; rag2^{E450fs} (casper)*	i.m.	$5.0x10^5$	3	0	4 (trunk and head kidney)	7	8
	Tg(mitfa:BRAF^{V600E}); tp53$^{-/-}$; mitfa$^{-/-}$	*nacre*	*MiniCoopR:GFP*	1° transplant in *rag2^{E450fs} (casper)*	*flk1:mCherry; rag2^{E450fs} (casper)*	i.m.	$5.0x10^5$	5	1 (invasion along the spinal cord)	2 (trunk kidney)	8	8
	Tg(mitfa:BRAF^{V600E}); tp53$^{-/-}$; mitfa$^{-/-}$; alb$^{-/-}$	*nacre*	*MiniCoopR:GFP*	1° transplant in *rag2^{E450fs} (casper)*	*rag2^{E450fs} (casper)*	i.m.	$1.0x10^5$	12	0	2 [1 (muscle tissue adjacent to thymus); 1 (thymus)] (Fig. 5c-e)	14	14
					rag2^{E450fs} (casper)	r.o.		9	3 [2 (tumour invades through the cranial cavity to spread into	0	12	15

									gills, further reaching to head and trunk kidney); 1(thymus)]			
				6° transplant in $rag2^{E450fs}$ *(casper)*	$rag2^{E450fs}$ *(casper)*	i.m.	$1.0x10^5$	4	0	0	4	5
					flk1:mCherry; $rag2^{E450fs}$ *(casper)*		$5.0x10^5$	1	1 (trunk kidney)	3 [1 (head kidney); 1 (region above the trunk kidney); 1 (scattered throughout the whole body)]	5	5
				7° transplant in $rag2^{E450fs}$ *(casper)*	$rag2^{E450fs}$ *(casper)*	i.m.	$5.0x10^5$	3	2 (invasion along the spinal cord)	0	5	5
					flk1:mCherry; $rag2^{E450fs}$ *(casper)*	i.m.	$5.0x10^5$	1	1 (invasion along the spinal cord)	3 [1 (head kidney); 1 (trunk and head kidney); 1 (thymus)]	5	5
	Tg(mitfa:BRAF^{V600E}*);* $tp53^{-/-}$*; mitfa*$^{-/-}$	*nacre*	*MiniCoopR:GFP*	1° transplant in $rag2^{E450fs}$ *(casper)*	$rag2^{E450fs}$ *(casper)*	i.m.	$5.0x10^5$	6	1 (invasion along the spinal cord, involving mesonephros)	0 (Fig. 6a)	7	7
					$rag2^{E450fs}$ *(casper)*	r.o.	$5.0x10^5$	3	12 [11 (tumour invades through the cranial cavity and spread into gills, further reaching to pronephros and mesonephros); 1 (tumour invades into the optic tectum/brain region)]	0	15	17
				5° transplant in $rag2^{E450fs}$ *(casper)*	$rag2^{E450fs}$ *(casper)*	i.m.	$5.0x10^5$	0	0	3 [2 (head kidney); 1 (muscle tissue beneath trunk kidney)]	3	3
				7° transplant in $rag2^{E450fs}$ *(casper)*	$rag2^{E450fs}$ *(casper)*	i.m.	$5.0x10^5$	0	0	4 [2 (kidney, then disseminate to whole body); 1 (trunk kidney); 1 (region above	4	4

										the trunk kidney)] (Fig. 6b,d)		
	Tg(mitfa:BRAF^{V600E}*); tp53*$^{-/-}$*; mitfa*$^{-/-}$	*nacre*	*MiniCoopR:GFP*	1° transplant in *rag2*^{E450fs} *(casper)*	*rag2*^{E450fs} *(casper)*	i.m.	3.5x10^{5}	14	1 (region above the trunk kidney)	0 (Supplementary Fig. 5a)	15	15
						r.o.	3.5x10^{5}	3	10 [5 (invasion into cranial cavity); 1 (invasion into cranial cavity, spreading into the gills); 3 (tumour invades through the cranial cavity to spread into gills, further reaching to head and trunk kidney); 1 (tumour invades into the optic tectum (brain) region)] (Fig. 5a)	0	13	15
				4° transplant in *rag2*^{E450fs} *(casper)*	*rag2*^{E450fs} *(casper)*	i.m.	5.0x10^{5}	3	0	0	3	3
				8° transplant in *rag2*^{E450fs} *(casper)*	*flk1:mCherry; rag2*^{E450fs} *(casper)*	i.m.	5.0x10^{5}	4	1 (whole kidney)	0 (Supplementary Fig. 5b)	5	5
